# Assessing the Readability and Quality of Web-Based Resources on Exercise Stress Testing: Cross-Sectional Readability and Quality Analysis

**DOI:** 10.2196/68000

**Published:** 2025-10-22

**Authors:** Munir Rahbe, Dhrumi Mistry, Ali M Khawaja, Natalie A Sous, Alan Y Tso

**Affiliations:** 1 Department of Medicine, Rutgers New Jersey Medical School, 185 S Orange Ave, Newark, NJ, 07103, United States, 1 (732) 445-4636; 2 Rutgers New Jersey Medical School, Newark, NJ, United States

**Keywords:** exercise stress test, readability, quality, Flesch-Kincaid grade level, Flesch reading ease, Simple Measure of Gobbledygook, Gunning Fog index, FKGL, FRE, SMOG, GF, DISCERN, mDISCERN, exercise, physical activity, cross-sectional study, quality analysis, health literacy, patient outcomes, search engines, websites, medical information, health information

## Abstract

**Background:**

As internet usage continues to rise, an increasing number of individuals rely on online resources for health-related information. However, prior research has shown that much of this information is written at a reading level exceeding national recommendation, which may hinder patient comprehension and decision-making. The American Medical Association (AMA) recommends that patient-directed health materials be written at or below a 6th-grade reading level to ensure accessibility and promote health literacy. Despite these guidelines, studies indicate that many online health resources fail to meet this standard. The exercise stress test is a widely used diagnostic tool in cardiovascular medicine, yet no prior studies have assessed the readability and quality of online health information specific to this topic.

**Objective:**

This study aimed to evaluate the readability and quality of online resources on exercise stress testing and compare these metrics between academic and non-academic sources.

**Methods:**

A cross-sectional readability and quality analysis was conducted using Google and Bing to identify web-based patient resources related to exercise stress testing. Eighteen relevant websites were categorized as academic (n=7) or nonacademic (n=11). Readability was assessed using four established readability formulas: Flesch-Kincaid Grade Level (FKGL), Flesch Reading Ease (FRE), Simple Measure of Gobbledygook (SMOG), and Gunning Fog (GF). Website quality and reliability were evaluated using the modified DISCERN (mDISCERN) tool. Statistical comparisons between academic and nonacademic sources were performed using independent samples *t* tests.

**Results:**

The average FKGL, SMOG, and GF scores for all websites were 8.36, 8.28, and 10.14, respectively, exceeding the AMA-recommended 6th-grade reading level. Academic sources had significantly higher FKGL (9.1 vs. 7.9, *P*=.03), SMOG (8.9 vs. 7.9, *P*=.04), and lower FRE scores (57.6 vs. 65.3, *P*=.006) than nonacademic sources, indicating greater reading difficulty. The average GF scores for academic and nonacademic sources were 10.68 and 9.81, respectively, but this difference was not statistically significant. The quality of web resources, as assessed by mDISCERN, was classified as fair overall, with an average score of 29.44 out of 40 (74%). While academic and nonacademic websites had similar mDISCERN scores, areas such as source citation, publication dates, and acknowledgment of uncertainty were consistently lacking across all resources.

**Conclusions:**

Online resources on exercise stress testing are, on average, written at a reading level that exceeds the AMA’s 6th-grade reading guideline, potentially limiting patient comprehension. Academic sources are significantly more difficult to read than nonacademic sources, though neither category meets the recommended readability standards. The quality of web-based resources was found to be fair but could be improved by ensuring transparency in sourcing and providing clearer, more comprehensive information. These findings underscore the need for improved accessibility and readability in online health information to support patient education and informed decision-making.

## Introduction

As of 2023, nearly 95% of adults in the United States use the internet [1]. In today’s information age, the challenge has shifted from simply accessing the internet to finding information that is both reliable and accessible. This shift is especially significant as more individuals rely on the internet for crucial health information. In 2022, 58.5% of adults reported using the internet at least once in the past year [2], and 70% turned to the internet as their primary source for health-related inquiries [3]. However, medical information is often nuanced, complex, and difficult to comprehend. What happens when patients encounter content that is difficult to read? Research shows that low health literacy is a significant independent predictor of all-cause mortality [4,5]. This emphasizes the vital role of the internet in modern health care and highlights the need for accessible, understandable health information to improve patient outcomes.

As early as 2000, professionals began publishing articles urging physicians to guide their patients using objective scales to “grade” the quality of online health information. The suggestion was that physicians should take responsibility for advising patients on how to “surf the net safely” when seeking health-related information [6]. Since then, the readability and quality of online health content accessed by patients have remained a key concern for clinicians, leading to numerous studies on the topic [7-10]. These studies examine various factors, but one of the most consistent is readability—specifically, the grade level at which the material is written. According to the American Medical Association (AMA), health materials should be written at or below a 6th-grade reading level to ensure appropriate health literacy for today’s patients [11].

The exercise stress test is a widely used diagnostic tool in the outpatient setting for individuals with potential coronary artery disease. It is also a valuable tool in the risk stratification of patients undergoing surgical procedures. The results of these tests have good prognostic value and essential information for clinicians [12]. Numerous studies evaluating the readability and quality of cardiovascular health topics have found that much of the available information is written at a reading level that exceeds recommended standards [13-15]. However, no studies to date have explicitly focused on exercise stress testing. This study will be the first to assess the readability and quality of online resources related to exercise stress testing.

The goals of this study are to (1) evaluate the readability and quality of health information on exercise stress tests and (2) compare these metrics between academic and nonacademic online resources.

## Methods

### Data Collection

To objectively assess the quality of available information for patients regarding cardiovascular exercise stress testing, the Google and Bing search engines were accessed. These search engines are ranked the top two respective search engines globally, accounting for approximately 94% of all search engine traffic [16]. The engines were interrogated using the four terms: “exercise stress test,” “exercise ECG,” “stress ECG,” and “treadmill ECG” on August 28, 2024. Searches were conducted on a nonuniversity affiliated internet connection in the respective engine’s incognito mode to simulate the experience of a default user. Analysis of relevant sites was limited to the first page of produced results in accordance with previous findings indicating that approximately 92% of all search engine traffic fails to extend beyond the first page [17].

As of August 2024, the Google search engine accounted for approximately 90.5% of global search engine traffic [16]. Additionally, we sought to include the Bing search engine, accounting for an additional 3.9% of search engine traffic, to assess a broader scope of patient experiences. Searches were conducted, and relevant sites were compiled; results were excluded if they were published in academic journals, published in academic textbooks, were written in a non-English language, were redundant with previous search results, were outside of the scope of the cardiac stress testing assessment, redirected to a nonarticle link, or promoted the sale of a product or service. These criteria were established to analyze the most relevant information a patient may encounter in researching this assessment that they would likely evaluate. The final analysis of results included eighteen web pages. The compiled results were categorized as academic (n=7) or nonacademic (n=11) and assessed for readability and quality. Academic results were operationally defined as sites affiliated with an academic institution, whereas nonacademic results were defined as publicly available websites containing relevant information not affiliated with an academic institution.

### Assessment of Readability and Quality

The Flesch-Kincaid Grade Level (FKGL), Flesch Reading Ease (FRE), Simple Measure of Gobbledygook (SMOG), and Gunning Fog (GF) tools were used to objectively assess the readability of internet-based resources pertaining to cardiovascular exercise stress testing. A modified DISCERN criterion (mDISCERN) was used to characterize the objective quality and reliability of the internet-based resource.

The Flesch-Kincaid Reading Ease (FRE) and Flesch-Kincaid Grade Level (FKGL) are the most widely used readability tools in health care literature [18]. These tools were originally developed in the mid-1900s for the United States Navy and were found to correlate well with the reading levels of sailors [19]. Since their inception, they have been adopted by various government organizations, including the Social Security Administration [20]. While these readability tools are not perfect, they are considered reliable when compared to other psychological tests used to assess reading level [21]. The FRE and FKGL indexes have been used extensively to objectively quantify the readability of health-related materials and are widely used in the literature for assessing the accessibility of health care information [8,9]. The FRE and FKGL metrics use mathematical formulas to quantify an objective readability score of written material based on the number of words per sentence and syllables per word [22,23]. FRE scores range from 0‐100, with greater scores indicating greater ease of reading material and lower scores indicating greater difficulty. The FKGL index uses the FRE score and extrapolates the produced value to a respective reading grade level. The FKGL scores use the same scale as the FRE index, with scores 80‐90 indicating a 6th-grade reading level and scores 0‐30 indicating a college reading level.

Despite the frequency of their use, the FRE and FKGL indices have noted variability in their accuracy with respect to particular literary resources [24]. To address any potential limitations of these tools, this study used two additional readability metrics commonly used in the health care literature—the Gunning Fog (GF) and Simple Measure of Gobbledygook (SMOG) assessments. Developed in 1952 and 1969, respectively, these tools were developed after the FRE and serve as additional commonly utilized readability metrics in evaluating online patient-directed health resources [25,26].

Although differences in the frequency of their use exist, the GF and SMOG indices have been noted to produce outputs that are more precise in evaluating the readability of written health information due to their modified criteria compared to the FRE and FKGL indexes [18]. Furthermore, the SMOG index has been indicated as the gold standard with respect to the readability of health-related resources due to its precision in scoring outputs and the recency of its development in relation to other tools [18,27]. The GF index uses a mathematical formula that evaluates the number of words, sentences, and complex (multi-syllabic) words to calculate an objective score that equates to the readability of a given text [28]. The later-developed SMOG index uses a modified mathematical model that also uses the number of complex words and sentences to generate an objective readability score. Similar to the FKGL, both metrics use the same scale, and the generated numerical score is designed to directly equate to the grade level required to comprehend the respective text.

Readability results across the FRE, FKGL, FOG, and SMOG indexes were calculated for each of the 18 compiled internet-based resources, and the respective scores were compared to the AMA guidelines, which recommend patient-targeted reading material be written at or below a 6th-grade reading level [11].

The DISCERN instrument, another widely used tool in the literature, has been validated for the evaluation of health-related information by both healthcare professionals and patients [29]. This instrument was used to objectively assess the quality of internet-based information regarding cardiac exercise stress tests [9,10]. The questionnaire consists of three sections with a total of sixteen questions, designed to evaluate both the reliability and overall quality of online health information. The first component of the instrument, questions 1 through 8, characterizes the reliability of a published resource. The second component, questions 9 through 15, characterizes the quality of treatment-related information. The third component, question 16, uses the compiled scores from the previous 15 questions to characterize how well the resource provides information regarding specific treatment options [30]. All questions are scored on a scale ranging from 1 to 5, with lower scores indicating poorer rankings and higher scores indicating more positive rankings. For the purposes of this study, a modified version` of the DISCERN instrument (mDISCERN) was used, consisting of only the first component of the tool, as this study solely concentrated on information surrounding diagnostic assessment and not subsequent treatment (Table 1). A mDISCERN score below 40% classified the reliability of a web result as “poor,” scores between 40% and 79% were deemed “fair,” and scores above 80% were categorized as “good.” These thresholds align with scoring criteria established in prior research studies [26,31].

**Table 1.** Modified DISCERN (mDISCERN) scoring tool.

#	Question	Score
1	Are the aims of the article clear?	1‐5
2	Does the article achieve stated aims?	1‐5
3	Are the topics covered relevant?	1‐5
4	Is it clear what sources of information were used to compile the publication other than the author?	1‐5
5	Is it clear when the information used or reported in the publication was produced?	1‐5
6	Is it balanced and unbiased?	1‐5
7	Does it provide details of additional sources of support and information?	1‐5
8	Does it refer to areas of uncertainty?	1‐5

Text from each of the eighteen compiled internet articles was copied into a Microsoft Word document. Image-derived text within the article was only included in the document if the text was present on a relevant graphic within the article and was clearly intended for the viewer to read. Two independent reviewers used the Microsoft grammar assist tool to calculate the FRE score and FKGL using their respective equations [22,23]. SMOG and GF scores were calculated by inputting identical website text used for FRE and FKGL scores into a widely used free online readability calculator [32]. The reviewers also used the mDISCERN criteria to evaluate each source material. Scores for each of the questions of the mDISCERN criteria were independently calculated, and scores were averaged.

### Statistical Analysis

Independent samples *t* tests were performed between mDISCERN, individual mDISCERN questions, FRE, FKGL, SMOG and GF scores of academic (n=7) and nonacademic (n=11) resources to distinguish any significant differences in the quality and readability of information based on the publisher. An alpha value of *P*=.05 was used to determine statistical significance for all performed analyses. All analysis was conducted on version 29.0.2.0 of the SPSS platform.

### Ethical Considerations

This study was exempt from review by the Rutgers University Office for Research Institutional Review Board as it was deemed Non-Human Subject Research (Pro2025001840). All information obtained over the course of the study is publicly available. All data, usernames, screenshots, and pictures used in this manuscript have been deidentified.

## Results

**Figure 1.**
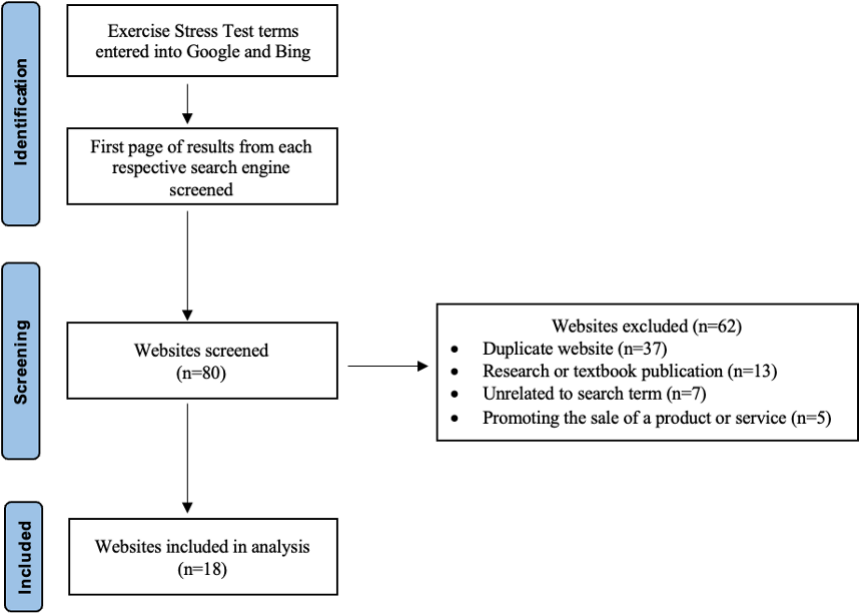
Screening methodology and exclusion criteria for website analysis.

Each search term was queried in two search engines, yielding a total of 80 websites. Of these, 62 were textbook chapters, duplicates, advertisement links, journal publications, or outside the scope of exercise stress testing. These results were subsequently excluded, and the remaining 18 websites were evaluated. Our screening process for the websites included in our evaluation is depicted in Figure 1. The websites were categorized into two groups: academic (n=7) and nonacademic (n=11). Results for the analyzed websites are available in Table 2. The FKGL ranged from 5.15 to 9.85; the mean score was 8.36 (SD 1.32). The mean FRE score of all the websites included in this study was 62.31 (SD 6.65). The mDISCERN scores ranged from 22‐36, with the mean being 29.44 (SD 3.98).

**Table 2.** Calculated metrics for academic (1-7) and nonacademic (8-18) sources.

Website^a^	FKGL^b^	SMOG^c^	GF^d^	FRE^e^	mDISCERN^f^
Cleveland Clinic [33]	8.8	8.7	11.4	53.1	36.0
Mayo Clinic [34]	7.5	8.5	8.5	61.7	36.0
Harvard Health [35]	9.0	8.9	10.7	60.0	27.5
Harvard Health [36]	9.8	9.7	11.6	56.4	27.0
Harvard Health [37]	9.2	9.3	10.8	60.5	29.5
Hopkins Medicine [38]	8.9	8.2	10.6	58.9	27.0
Massachusetts General Hospital [39]	10.4	9.0	11.1	52.5	28.0
American Heart Association [40]	8.1	8.7	11.0	62.4	22.0
St. Vincent’s Heart Health [41]	8.6	8.6	11.4	61.3	26.5
Medline Plus [42]	7.8	7.3	9.3	64.2	36.0
WebMD [43]	6.7	7.8	9.7	70.1	29.5
Healthline [44]	8.4	7.3	9.0	62.7	33.5
British Heart Foundation [45]	6.7	6.8	8.6	73.3	29.5
Heart and Stroke Foundation Canada [46]	9.0	9.5	11.3	58.4	27.5
MyHealth Alberta [47]	5.2	5.3	6.4	77.7	29.5
HeartWest [48]	9.9	9.2	11.0	60.1	29.5
SoZo Cardiology [49]	9.5	9.5	12.1	57.5	28.0
Ascot Cardiology [50]	7.0	6.8	8.1	70.8	27.5

a Academic sources (1-7) and nonacademic (8-18) sources.

b FKGL: Flesch-Kincaid grade level.

c SMOG: Simple measure of gobbledygook.

d GF: Gunning Fog.

e FRE: Flesch reading ease.

f mDISCERN: modified DISCERN.

**Figure 2.**
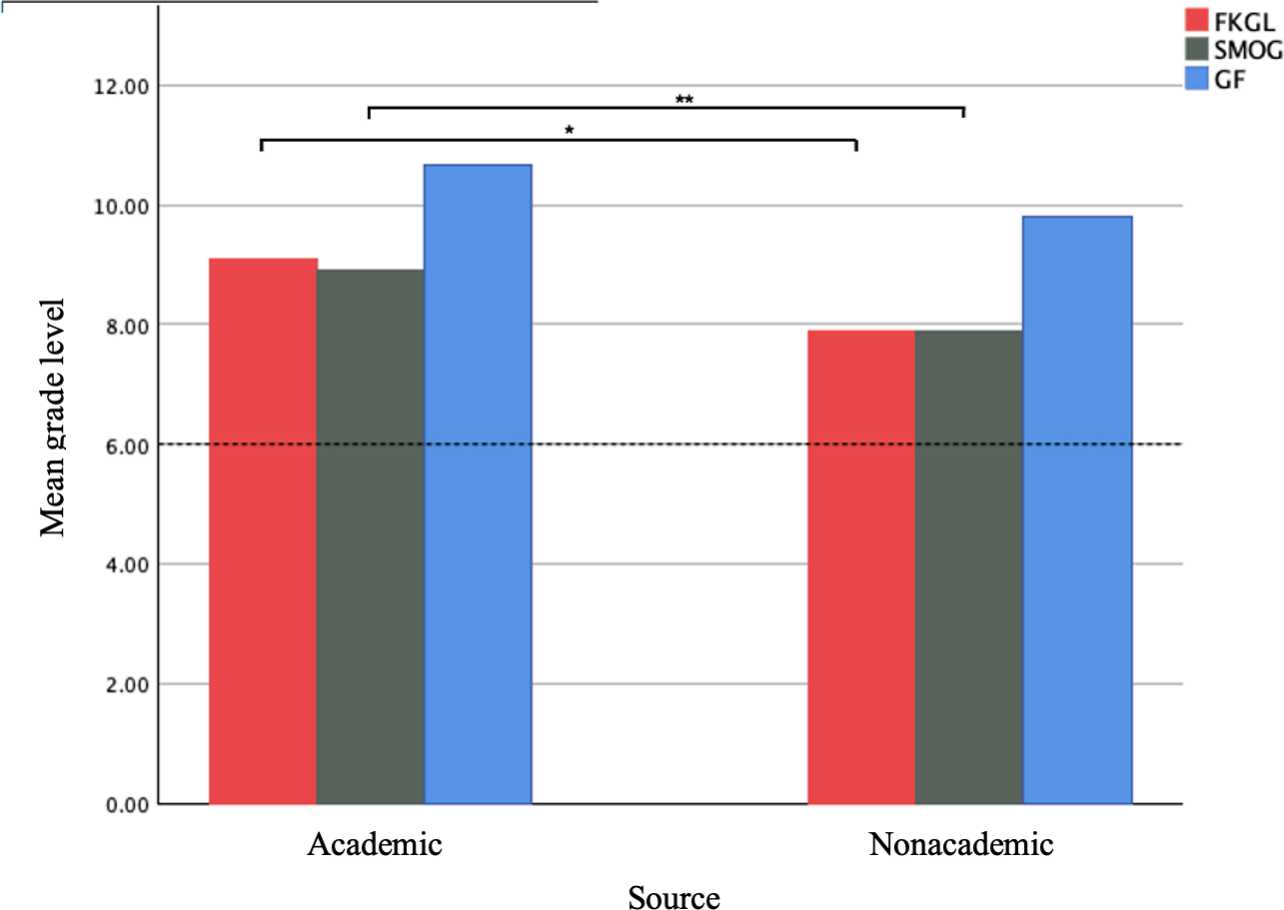
Mean FKGL, SMOG, and GF of academic and nonacademic sources. The dotted red line represents the American Medical Association’s recommended 6th-grade reading level. FKGL: Flesch-Kincaid grade level; GF: Gunning Fog; SMOG: Simple Measure of Gobbledygook. *P=.03 **P=.04.

**Figure 3.**
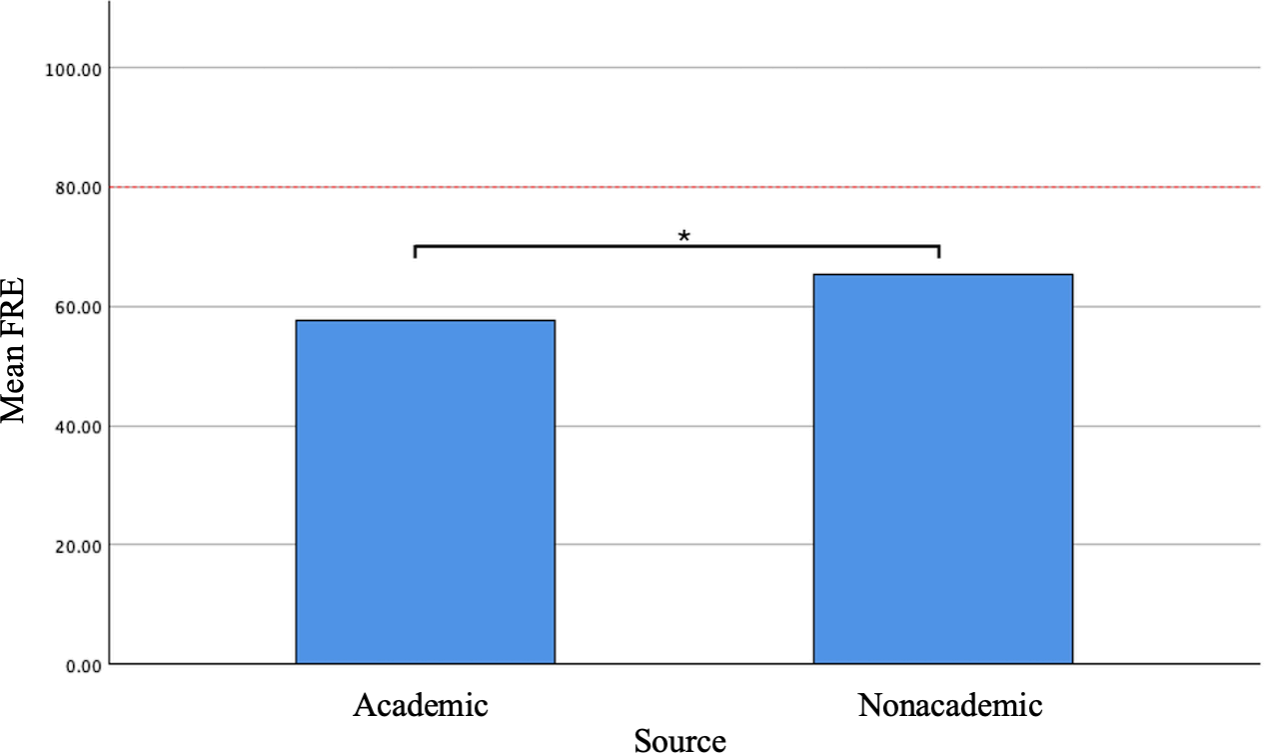
Mean FRE of academic and nonacademic sources. The dotted red line represents the equivalent FRE score for a 6th-grade reading level. FRE: Flesch Reading Ease. *P=.006

When evaluating by website type, academic websites had a greater mean FKGL (9.09, SD 0.90 vs 7.90, SD 1.40), SMOG (8.90, SD 0.50 vs 7.90, SD 1.34), and GF (10.68, SD 1.03 vs 9.81, SD 1.73) than nonacademic websites. An independent samples *t* test found the mean FKGL and SMOG between academic and nonacademic websites to be statistically significant, with a *P* value of .03 and .04, respectively. (Figure 2). Similarly, a statistically significant difference in the mean FRE for academic (57.6, SD 3.66) and nonacademic (65.32, SD 6.6) sources was found with a *P*=.006 (Figure 3). Academic (30.14, SD 4.18) and nonacademic (29, SD 3.88) sites had similar mDISCERN scores. For academic websites, mDISCERN scores ranged from 27‐36, while nonacademic websites mDISCERN scores ranged from 22‐36.

Among the 8 individual mDISCERN questions, question 1 had the greatest score amongst all the websites (4.89, SD 0.24). Academic sites (5, SD 0) had a greater score for question 1 compared to nonacademic sites (4.81 SD 0.50). Question 4 had the lowest score amongst all websites (1.83 SD 1.23). Academic sites (2 SD 1.30) had a greater score for question 4 versus nonacademic (1.73 SD 1.20) sites. Academic sites had a perfect score for question 2 as well, with a mean of 5 (SD 0).

## Discussion

### Principal Findings

Our study is the first to assess the readability and quality of web-based sources on exercise stress testing. These results indicate that the average FKGL, SMOG and GF scores for the 18 web pages analyzed were 8.36, 8.28, and 10.14, respectively. These average scores exceed the AMA’s recommendation that health information be written at a 6th-grade level [11]. Our analysis also shows that academic sources for health information are written at a significantly higher grade level than nonacademic sources, with average FKGL scores of 9.1 and 7.9, and SMOG scores of 8.9 and 7.9, respectively. While the grade level of academic sources is statistically greater, both categories exceed the AMA recommendation for health information to be written at a 6th-grade level, indicating that even nonacademic sources may still be too complex for the average patient to fully understand. Of the 18 websites analyzed, none met the recommended reading level across all three scoring criteria. Only one source met the criteria for two readability metrics with a FKGL of 5.2, SMOG of 5.3, and GF of 6.4

As the FRE and FKGL formulae depend on many of the same parameters, it follows that the average FRE for academic sources was statistically lower than for nonacademic sources. An FRE score of 80‐90 is considered appropriate for a 6th-grade reading level [22,23]. In contrast, the average FRE scores for academic and nonacademic sources were 57.6 and 65.3, respectively. Although this difference was statistically significant, both scores indicate reading difficulty levels that exceed the recommended ease of 80‐90.

Readability was further analyzed using the SMOG and GF tools. We chose to include these readability metrics for a more thorough analysis of the websites in this study. These tools are highly regarded in the assessment of health care literature and are more modern readability models based on the initial Flesch formulas [18,27]. Scores of 6 or below on the SMOG and GF scales are considered to be in accordance with the AMA-recommended guidelines. The mean calculated SMOG scores for academic and nonacademic sources were 8.9 and 7.9, respectively. Similar to the findings from the Flesch scores, our analysis revealed statistically significant differences in the calculated SMOG scores between academic and nonacademic sources, with academic sources written at a significantly higher grade-level. Despite these differences, both source types exceeded the recommended reading level. The average GF scores for academic and nonacademic sources were 10.68 and 9.81. Although we found no significant difference between the scores between the source types, both scores indicated that the material is written at a reading level that exceeds the current recommendation.

These findings highlight that both academic and nonacademic institutions are producing resources on exercise stress tests that fail to meet current readability recommendations, potentially hindering patients’ ability to fully understand their health information. This lack of concordance with recommended readability standards may limit patients’ capacity to make informed decisions about their care, thereby undermining their participation in shared decision-making and their ability to manage their health effectively.

Our analysis also focused on the quality of web resources using the mDISCERN criteria. As described in our methods, web resources with a mDISCERN score below 40% classified the reliability as “poor.” Scores between 40% and 79% were deemed “fair,” and scores above 80% were categorized as “good.” The average mDISCERN score for all web resources included in our analysis was 29.44 out of 40 (74%). This classifies the average quality of web resources as “fair” and closer to “good” than “poor.” 14 of the web resources were rated as “fair,” and the remaining four were rated as “good.” Every web resource analyzed lost points when evaluated on questions four, five, seven, and eight. Questions four and five pertain to clearly citing the sources used in the article and providing publication dates for both the article and its references. Questions 7 and 8 assess whether the author offers resources for further information and addresses potential uncertainties, such as the risks associated with the test. We considered these areas crucial for assessment, as they were consistently lacking across all the web resources reviewed. By addressing these deficiencies, the overall quality of these resources can be significantly enhanced, providing patients with more reliable and comprehensive information.

The available web resources for patients seeking information on exercise stress tests are, on average, of fair quality but exceed the recommended reading level when assessed using the FKGL, FRE, SMOG and GF scores. These findings are consistent with other studies analyzing the readability of cardiovascular topics [13-15], suggesting that many online health resources related to cardiovascular medicine may be difficult for the average patient to understand, potentially limiting their accessibility and effectiveness in promoting patient education. Authors of both academic and nonacademic resources could improve their content by consulting the AMA guidelines on best practices for creating patient-friendly health information [11]. In particular, attention should be given to factors that reduce reading ease and elevate the reading grade level.

### Limitations

As previously mentioned, the FKGL and FRE are the most widely used readability formulas in health care literature [18]. To enhance the comprehensiveness of our analysis, we incorporated the SMOG and GF readability metrics as additional objective tools for evaluating website readability. While the Flesch formulas remain the most commonly used in health care, the SMOG and GF metrics provide more modern and potentially more relevant approaches for the assessment of health-related information [18,27]. However, these formulas have inherent limitations, as they rely primarily on quantifiable factors such as syllable count, word count, sentence count, and sentence length [22-24,28]. These tools are not capable of assessing the complexity or technical nature of health-related information. Regardless of sentence or syllable count, short sentences composed of mono- or bi-syllabic words may still contain complex medical terminology that is difficult for the reader to understand, potentially leading these formulas to underestimate the actual reading level required for comprehension. Alternative language in health-related information can be used when appropriate; however, avoiding vital terminology to lower the reading grade level may compromise the accuracy and clarity of the information provided. Simplifying medical jargon might increase readability, but at the potential cost of misinforming patients.

Additional limitations arise while using the DISCERN tool for evaluating articles. Scoring is inherently subjective, requiring an evaluator to assess a web page and rate each question accordingly. While this nonformulaic approach allows for a more nuanced evaluation, individual evaluator bias and interpretation can affect the consistency and standardization of scores.

### Conclusion

The web resources available to patients seeking information regarding exercise stress tests are written at a reading level that surpasses the current AMA recommendation. This is complicated by the inherently complex nature of medical terminology. Despite this, efforts should be made by authors to use alternative terms or omit terms altogether if the medical jargon being used does not contribute to the message attempting to be conveyed. The quality of the web resources evaluated were determined to be fair but could be improved by providing more comprehensive information. While improving health literacy across the population is crucial, it is equally important to provide patients with clear, accessible materials that empower them to make informed decisions about their health. These materials must also be high-quality, offering relevant, unbiased, and concise information, along with appropriate access to sources and additional resources for those seeking further guidance.

## Abbreviations

AMA: American Medical Association
FKGL: Flesch-Kincaid Grade Level
FRE: Flesch Reading Ease
GF: Gunning Fog
mDISCERN: modified DISCERN
SMOG: Simple Measure of Gobbledygook
